# Patch clamp recordings of action potentials from pyramidal neuron in hippocampus CA1 under focused ultrasound neurostimulation with MEMS self-focusing acoustic transducer

**DOI:** 10.1088/1741-2552/ade7ae

**Published:** 2025-07-04

**Authors:** Jaehoon Lee, Yongkui Tang, Akash Roy, Kianoush Sadeghian Esfahani, Su-Youne Chang, Eun S Kim

**Affiliations:** 1 Ming Hsieh Department of Electrical and Computer Engineering, University of Southern California, Los Angeles, CA, United States of America; 2 Department of Neurologic Surgery and the Department of Physiology and Biomedical Engineering, College of Medicine, Mayo Clinic, Rochester, MN, United States of America

**Keywords:** patch clamp, neuromodulation, focused ultrasound stimulation, acoustic transducer, microelectromechanical system, focused ultrasound neuromodulation, focused ultrasound neurostimulation

## Abstract

*Objective.* This study aims to investigate the modulatory effects of focused ultrasound (FUS) on neuronal activity at the single-cell level, using whole-cell patch clamp recordings in hippocampal slices. *Approach.* A self-focused acoustic transducer (SFAT) was designed and fabricated on a 127 *µ*m-thick translucent lead zirconate titanate substrate to allow infrared light transmission for visualizing neurons during patch clamp experiments. The SFAT operates at 18.4 MHz, producing low-intensity FUS with a 46 *µ*m focal diameter at a depth of 400 *µ*m. Three types of SFAT—active, FUS-blocking control, and low-electromagnetic interference (EMI) versions—were developed to assess the effects of acoustic stimulation, thermal heating, and EMI. Neuronal responses were recorded across 78 tissue samples from 29 animals using 48 combinations of acoustic parameters, including peak-to-peak voltage, pulse repetition frequency (PRF), and pulse duration. *Main results.* Whole-cell patch clamp recordings from CA1 pyramidal neurons in rat hippocampal slices revealed that FUS induces both inhibitory and excitatory effects on action potential firing, depending on the stimulation parameters. Inhibition was found to be the dominant response, while excitation was mainly attributable to thermal effects. Optimal inhibition was achieved with 60 Vpp (ISAPA = 2.11 W cm^−2^), 35 kCycles/pulse (1.90 ms), and 100 Hz PRF, yielding a 60% success rate. Conversely, excitation was observed in 60% of trials using 120 Vpp (ISAPA = 8.44 W cm^−2^), 50 kCycles/pulse (2.72 ms), and 20 Hz PRF. *Significance.* This work presents a novel neuromodulation platform that combines high-frequency focused ultrasound with real-time whole-cell patch clamp recording at single-neuron resolution. The results provide direct electrophysiological evidence of parameter-dependent, bidirectional modulation of neuronal activity by FUS, offering new insights into its underlying mechanisms and helping define stimulation protocols for future neurotherapeutic applications.

## 1. Introduction

On-demand neuromodulation can be used to treat neurological disorders such as Parkinson’s disease [1] and epilepsy [2] as well as to investigate how the brain works. Most clinical neuromodulation is based on implanted electrode probes and implanted pulse generators, necessitating neurosurgery for the implantation. In addition, the implanted probes have long-term reliability issues, leading to the ineffectiveness of the probes over time, as hardened tissues form around the probes. It has also been reported that implanted electrodes can cause the gradual loss of neurons within approximately 50–70 *μ*m distance from the microelectrodes [3]. Various alternative neurostimulation techniques have been explored, such as optical stimulation [4], chemical stimulation [5], transcranial magnetic stimulation (TMS) [6], transcranial electrical stimulation (TES) [7], thermal stimulation [8], and ultrasound stimulation [9]. Each of these techniques presents its own advantages and disadvantages.

Optical stimulation, also known as optogenetic stimulation, uses light-sensitive channels or opsin which can be virally introduced into neurons. Optical stimulation with optical fibers offers a spatial resolution of 0.1–1 mm and a temporal resolution in the order of milliseconds [10]. One notable advantage of optogenetics is its ability to provide cell-type-specific stimulation within a tissue through selective genetic activation and inactivation of light-sensitive ion channels [4]. However, optogenetic stimulation requires injection of viruses and has limited penetration depth [11].

The chemogenetic method is similar to optogenetic in that it also requires genetic modifications of tissue, but employs chemically engineered molecules or ligands instead of light or light-sensitive channels. Chemical stimulation commonly involves designer receptor exclusively activated by designer drugs to ensure a good spatial selectivity [12], but suffers a slow temporal resolution of minutes–hours [13]. Though both optical and chemical neuromodulations have been useful in understanding brain activity, they share a limitation in requiring genetic modification of the tissue, which hinders their translation to clinical applications for which electrode implantation remains the preferred method [14].

TES and TMS are non-invasive neuromodulation techniques using electromagnetic waves and magnetic fields, respectively. However, the efficacy of the current delivered to neurons through TES is not as potent as that by TMS in eliciting an action potential (AP) [15]. Additionally, TES faces limitations in spatial resolution, though TMS can be focused on a small volume. Prefrontal TMS therapy is widely employed for treating major depressive disorder by depolarizing cerebral neurons, and it has received approval from the US Food and Drug Administration (FDA) [16]. Despite being considered a safe tool, TMS requires a large coil to generate the magnetic field, which is attached to the patient’s head and may be accompanied by a few side effects such as recurrent headaches and a tingling sensation on the face [17].

In contrast, focused ultrasound stimulation (FUS) offers spatial focusing as small as 10 *µ*m in diameter [18], with a temporal resolution as fast as 1.5 ms [19]. Unlike magnetic stimulation, FUS does not necessitate large equipment to deliver sufficient acoustic energy for eliciting neuronal activities; the drive electronics can be miniaturized and made portable. Leveraging these advantages, FUS is used in clinical settings for imaging and tumor ablation [20]. Moreover, the FDA has recently approved a FUS device for treating patients with advanced PD who experience symptoms such as mobility issues, rigidity, or dyskinesia [21]. This underscores the growing acceptance and application of FUS in therapeutic interventions.

In 1958 Fry *et al* reported a temporary neural inhibition in a cat’s eye when it was stimulated with a focused ultrasonic beam onto the lateral geniculate nuclei [22]. Since then, ample efforts have been made for FUS neural modulation in both the central and peripheral nervous systems. Some studies indicate that low-intensity ultrasound (*I*_SPTA_ = 23 mW cm^−2^, *I*_SPPA_ = 2.9 W cm^−2^) at low frequencies (0.44–0.66 MHz) can increase Na^+^ and Ca^2+^ currents [9]. Transcranial pulsed ultrasound (*I*_SPTA_ = 84.32 mW cm^−2^, *f*= 0.25 MHz) has been reported to increase spike frequency by triggering TTX-sensitive neuronal activity without a significant rise in brain temperature (<0.01 °C) [23]. Moreover, continuous ultrasonic stimulation at 43 MHz for 200 ms is reported to activate Piezo1 channels in human embryonic kidney cells [24], as well as TWIK-related acid-sensitive K+ (TASK) channels in pyramidal cells of the hippocampus CA1 region [25]. Applying 10 MHz ultrasound (*I*_SPTA_ = 4.9 W cm^−2^) for 20 ms with a 1 kHz pulse repetition frequency (PRF) is reported to modulate and increase Nav1.5 channel current [26]. Furthermore, ultrasonic waves have been reported to alter the passive properties of cell membranes, including conductance and capacitance [25, 27]. More recent reports suggest that FUS can both stimulate and inhibit neurons through tuning the PRF and pulse duration [28–31].

Although FUS-based neural stimulation and inhibition have been widely reported at the behavioral level and molecular level, the underlying mechanism for such effects is still not well understood. Patch clamp experiment is a good technique that can be used to understand the mechanism of FUS-based neural stimulation, since it allows monitoring of neuron activities at a cellular level. But the whole-cell patch-clamp experiment requires a good visibility of cells on the top of a neural tissue with light shining from the beneath of the tissue with a FUS transducer in between the tissue and the light source, typically absorbing the light. Consequently, various approaches such as a blind patch clamp [31], usage of reflector cones to redirect acoustic waves [29], and emulation of acoustic effects with mechanical poking with a glass pipette [32] have been explored with limited effectiveness, as these methods fail to achieve the precise control of FUS on the target neuron. This paper describes the FUS neuromodulation study with patch clamp experiments using a self-focusing acoustic transducer (SFAT) built on a thin and translucent 127 *μ*m thick lead zirconate titanate (PZT) substrate. The microfabricated SFAT (with its center area having no electrode) has a small, planar footprint and good infra-red (IR) light transmittance, making it an excellent fit for whole-cell patch clamp experiments on rat brain slices. The translucent transducer allows IR light to pass through, ensuring good visibility of cells when patching a glass pipette onto a neuron cell on the brain tissue that sits directly on the transducer. With the SFAT operating at 18.4 MHz, we have successfully carried out 323 whole-cell patch clamp experiments on pyramidal neurons in the CA1 region of hippocampal slices from Sprague Dawley rats with a variety of pulsed FUS parameters such as peak-to-peak voltage, PRF, and pulse duration or the number of cycles per pulse. The experiment results indicate the bi-directional neuromodulation (inhibition and excitation) depending on the FUS parameters and the optimal parameters for both inhibition and excitation are reported in this paper.

## 2. Device design, fabrication, simulation and characterization

### 2.1. Device design

An SFAT is composed of an ultrasonic sound source responsible for the generation of ultrasound waves and a patterned electrode designed for near-field wave interference. The sound source is a bulk sheet of piezoelectric PZT, which is sandwiched by its top and bottom electrodes. A PZT-5A is chosen as a substrate piezoelectric material for its high piezoelectric coefficient and electromechanical coupling coefficient. When a sinusoidal electrical signal, whose frequency is matched with the thickness-mode resonance of a PZT thin substrate, is applied between the top and bottom electrodes, the PZT vibrates with its resonance and generates acoustic waves [33]. A 127 *µ*m PZT substrate, which offers good IR transparency, is chosen for a thickness-mode resonance frequency ${f_n}\,$ of about 18.4 MHz.

**Figure 1.**
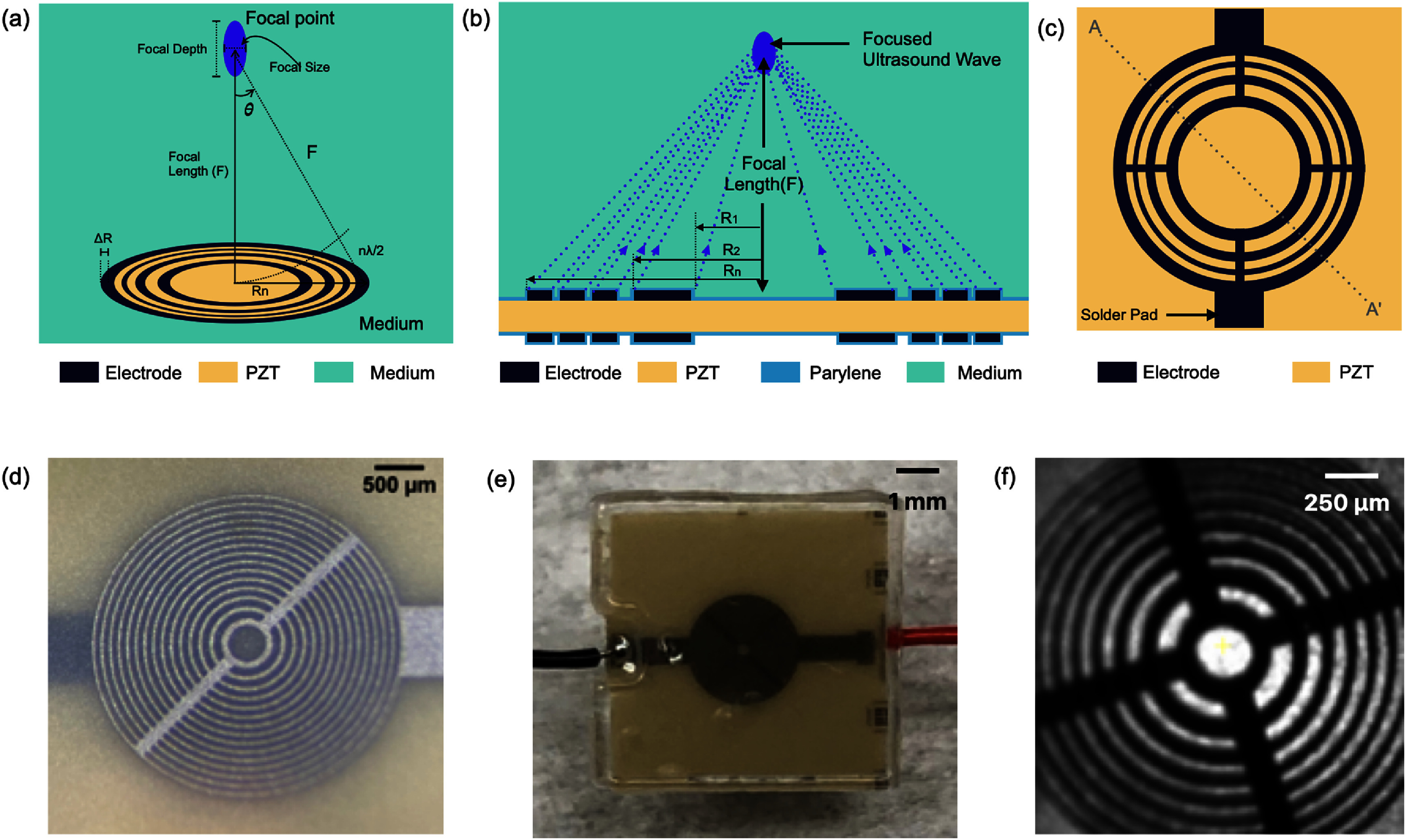
(a) Conceptual diagram of focused ultrasound generated by SFAT, defining the focal size, focal depth, focal length, half angle ($\theta $), ring radius (${R_n}$), and the width of the outermost ring (${{\Delta }}R$). (b) The cross-sectional diagram (across the AA′ dashed line in (c)) showing a typical SFAT based on a PZT substrate with patterned Fresnel annular-rings electrodes. This illustration highlights how the Fresnel acoustic lens focuses ultrasound in the medium by selectively letting constructively interfering acoustic waves arrive at the focal point in-phase. (c) The top-view illustration of the same SFAT, showing the patterned top electrode and solder pad. Top-view photos of (d) a fabricated SFAT for neuromodulation, (e) the bottom side of the SFAT with 0.5 mm thick polyester sheet attached, and (f) an SFAT placed in a patch-clamp setup with IR light illuminated from the bottom to show the translucent characteristic of the 127 *µ*m thick PZT.

Focusing is achieved by ensuring that the acoustic waves arrive in phase (0°–180°) at a focal point (at a distance *F* away from the transducer center) [34] through patterning the top and bottom electrodes into Fresnel half-wavelength band (FHWB) rings (figures 1(b) and (c)). When alternating current voltage of the resonant frequency is applied across the top and bottom electrodes, only the portions of PZT sandwiched between the electrodes vibrate and generate in-phase acoustic waves. Geometrically, each ring’s boundary is designed so that the distance to the focal point is equal to integer multiples of the half wavelength ($\lambda /2$) in the medium plus the focal length (*F*) (figure 1(a)). The radii of rings ${R_n}$ corresponding to a desired focal length (*F*) can be determined using the following equation

\begin{align*}{R_n} = \,\sqrt {n\,\lambda \, \times \left( {F + n\,\lambda /4} \right)} \,\,\,\,\left( {n = 0,\,1,\,2, \cdot \cdot \cdot } \right)\end{align*}

where $F$ and *λ* are focal length and wavelength, respectively.

Focal size, defined as the full width at half maximum of the acoustic pressure, created by the Fresnel annular-ring lens is approximately equal to the width of the outermost ring (${{\Delta }}{R_n}$) [35] and is:

\begin{equation*}{{\Delta }}R \approx \lambda F\,/2\sqrt {n\lambda F} = \sqrt {\lambda F/4n} = \sqrt {cF/4nf} \end{equation*}

where *c, F, n* and *f* are the sound velocity in the medium, focal length, number of rings in the Fresnel lens, and the operating frequency, respectively [36].

The design of FHWB rings can be made with a ‘positive source’ having a circular electrode in the center or a ‘negative source’ with no electrode in the center. For the patch clamp experiments, the negative source is used to allow light to pass through the SFAT center where there is no opaque electrode. The focal length is designed to be 400 *µ*m according to the thickness of a rat brain tissue prepared for the patch-clamp experiment. As the focal depth, defined as the vertical resolution of the focal zone (figure 1(a)), is approximately twice the lateral focal size in the SFAT design [36], the effective focal zone of the 400 *µ*m focal length design in the vertical direction is estimated to be around 350–450 *µ*m. This ensures that the ultrasonic waves can be focused near the top surface of the patched tissue, where the neuron cells are patched. The wavelength of the acoustic wave of 18.4 MHz in the brain tissue, where the speed of sound is approximately 1540 m s^−1^ [37], is about 84 *µ*m. The transducer is designed to have 15 constructive Fresnel rings for a focal size of around 46 *µ*m (equation (2)), resulting in the largest ring diameter of about 3.1 mm (figure 1(d)). The focal zone is aligned at the center of the SFAT in a lateral dimension, with the central opening (through which IR light passes) measuring 187 *µ*m in diameter (figures 1(c) and (f)). This configuration ensures that the only neurons located within the central opening are clearly visible during patch-clamp experiments, allowing precise targeting of neurons within the focal size of 46 *µ*m laterally, with the reduced risk of missing the target. Additionally, a 0.5 mm-thick polyester sheet is attached to the bottom of the SFAT to provide enhanced support and mechanical robustness during the experiments (figure 1(e)), followed by coating the surface with 28 *µ*m thick Parylene D as the acoustic matching and electrical insulation layer. figure 1(f) shows the translucent characteristic of SFAT when exposed to IR light emitted from the bottom of the transducer.

### 2.2. Simulation and measurement of SFAT

**Figure 2.**
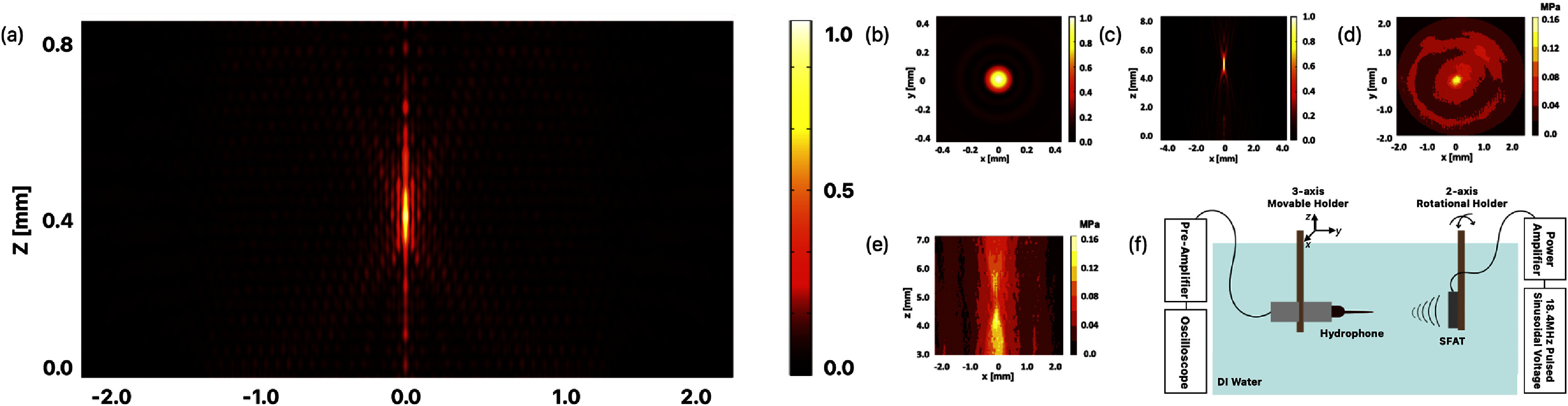
(a) Finite element analysis (FEA) of the normalized acoustic pressure distribution of the 15-ring SFAT at 18.4 MHz with a designed focal length of 400 *µ*m in the brain tissue media. The simulation result shows a focal size of 46 *µ*m and a focal depth of 95 *µ*m. (b)–(e) FEM simulations and measurements of the normalized acoustic pressure on the *z* = 5 plane and on the vertical plane. (f) Illustration of the hydrophone measurement setup in the scanning tank.

The distribution of acoustic pressure generated by an SFAT is simulated with finite-element analysis (FEA) [38] in the frequency domain using the Pressure Acoustics module of COMSOL Multiphysics 5.6 (COMSOL Inc.). The simulation is modeled with a two-dimensional axial symmetry to significantly reduce the computation time and memory, as SFAT has a symmetrical design. This approach allows modeling only half of the volume cross-section and the complete simulation results can be reconstructed by mirroring the simulated data along the central vertical axis (*R* = 0). For simplicity, only the volume of the medium above the transducer top surface where the actual acoustic waves propagate is considered. The vibration of the piezoelectric sheet is modeled as a normal displacement boundary condition applied only to the Fresnel annular-ring electrode areas. The simulation employs a free triangular mesh, with the maximum element size set to be $\lambda /5$ with *λ* being the wavelength of the acoustic wave [39]. Additionally, the propagating medium is assumed to be isotropic and homogeneous, and spherical wave radiation boundary conditions are applied at the outer boundaries of the simulation volume, where acoustic waves can pass through without reflection. Figure 2(a) shows the FEA simulation of the normalized acoustic pressure distribution in brain tissue, indicating the focal length, the focal size, and the focal depth of 390, 46, 95 *µ*m, respectively.

The acoustic pressure generated by the fabricated SFAT is measured using a needle hydrophone (HNP0200, Onda Corp.) with a tip diameter of 200 *µ*m in the scanning tank system (AIM III, Onda Corp.). To prevent the hydrophone measurement from being dominated by electromagnetic interference (EMI) when the hydrophone is too close to the transducer, the same SFAT with a longer focal length of 5 mm is fabricated and compared with the simulation result (figures 2(b)–(e)). A sinusoidal signal of 18.4 MHz is periodically delivered to the SFAT, consisting of only three cycles per pulse, at a PRF of 1 kHz during the hydrophone measurement to minimize the effect of EMI. The hydrophone is connected to a 20 dB pre-amplifier (AH-2010, Onda Corp.) and is securely fixed to a three-axis precision moveable stage. The SFAT is attached to a two-rotational-axis stage to align the beam axis between the hydrophone and the SFAT center (figure 2(f)). The acoustic signals picked up by the hydrophone are visualized with a digital oscilloscope (Picoscope 5000, Pico Technology Inc.), and the root-means-square voltage is converted into the peak acoustic pressure. The measured peak acoustic pressure, *P*_0_, at the focal point is used for calculating the spatial peak pulsed average acoustic intensity, ${I_{{\text{SPPA}}}}$ [40],

\begin{equation*}{I_{\text{SPPA}}} = \,\frac{{P_0^2}}{{2\rho c}}\end{equation*}

where $\rho \,$ and $c$ are the density of the medium and the speed of sound in the medium, respectively. By multiplying *I*_SPPA_ with a PRF over the pulse duration, the Spatial Peak Time Average acoustic intensity, *I*_SPTA_, is calculated:

\begin{equation*}{I_{{\text{SPTA}}}} = \mathop {{{\mathop \int \limits}}}\limits_0^T {I_{{\text{SPPA}}}}{\text{d}}t \times {\text{PRF}}{\text{ }}.\end{equation*}

The measurement results with 40*V*_pp_ applied to SFAT match the simulation results, and the SFAT is estimated to generate an intensity of 0.16 MPa at the focal point when actuated with 40*V*_pp_.

With the use of a 200 *µ*m-diameter needle hydrophone, larger than the transducer’s focal diameter of 46 *µ*m, the acoustic pressure measurements are subject to spatial averaging effects. As a result, the reported intensities reflect the spatial average pulse average (*I*_SAPA_) and the spatial average time average (*I*_SATA_) rather than the spatial peak pulse average (*I*_SPPA_) and the spatial peak time average (*I*_SPTA_). Thus, *I*_SAPA_ and *I*_SATA_ are used throughout the paper to describe the FUS parameters.

### 2.3. Low-EMI SFAT

Acoustic transducers not only generate acoustic waves but also emit EMI energy into the surrounding medium. While this EMI is generally not problematic for most applications, it can pose an issue when recording electrical signals from neurons immersed in the same medium and positioned above the SFAT. Given that the amplitude of the AP generated by neurons is approximately 100 mV, it is crucial to control and minimize the EMI induced by SFAT to prevent it from overwhelming the AP signals. Typically, tissues act as EMI absorbers, absorbing EMI much more effectively than water [41].

When the size of brain tissue is large, it effectively absorbs most of the EMI. However, in the case of a smaller tissue size, typically prepared from a young mouse, the tissue may not absorb enough EMI, leading to the dominance of EMI in the electrical recording. To tackle the EMI issue, a low-EMI SFAT is developed by depositing an additional metal layer on top of the SFAT. Any metal serves as an effective EMI shielding material to block EMI generated from the transducer since electromagnetic waves propagating through metal attenuate very rapidly, as the amplitude *A* of EMI at traveling inside a metal can be expressed as

\begin{equation*}A\left( d \right) = {A_0}{{\text{e}}^{ - d/\delta }}\end{equation*}

where ${A_0},d$, and $\delta $ are the initial amplitude, traveling distance, and skin depth of the metal, respectively. The amplitude of EMI is reduced to approximately 37% at the traveling distance of the skin depth, which can be obtained from

\begin{equation*}\delta \, = \sqrt {\frac{{2\rho \,\,}}{{\omega \,{\mu _r}{\mu _0}\,}}} \,\end{equation*}

where $\rho ,\,{\mu _r},{\mu _0}$ and $\omega $ are the resistivity, relative permeability of the metal, permeability of vacuum (${\mu _0} = 4\pi \, \times {10^{ - 7\,}}\,{\text{H}}\;{{\text{m}}^{ - 1}}$), and angular frequency ($\omega = 2\pi f$), respectively. Aluminum (Al), Copper (Cu), and Nickel (Ni) are commonly used for EMI shielding [42], and their material properties along with skin depths at a frequency of 18.4 MHz are listed in table 1.

**Table 1.** Material properties and skin depths of aluminum, copper, and nickel.

Metals	Aluminum	Copper	Nickel
Resistivity (*µ*Ω cm)	2.65	1.68	6.84
Relative permeability	1.00	0.99	600
Skin depth (*µ*m)	19.12	15.20	1.25

**Figure 3.**
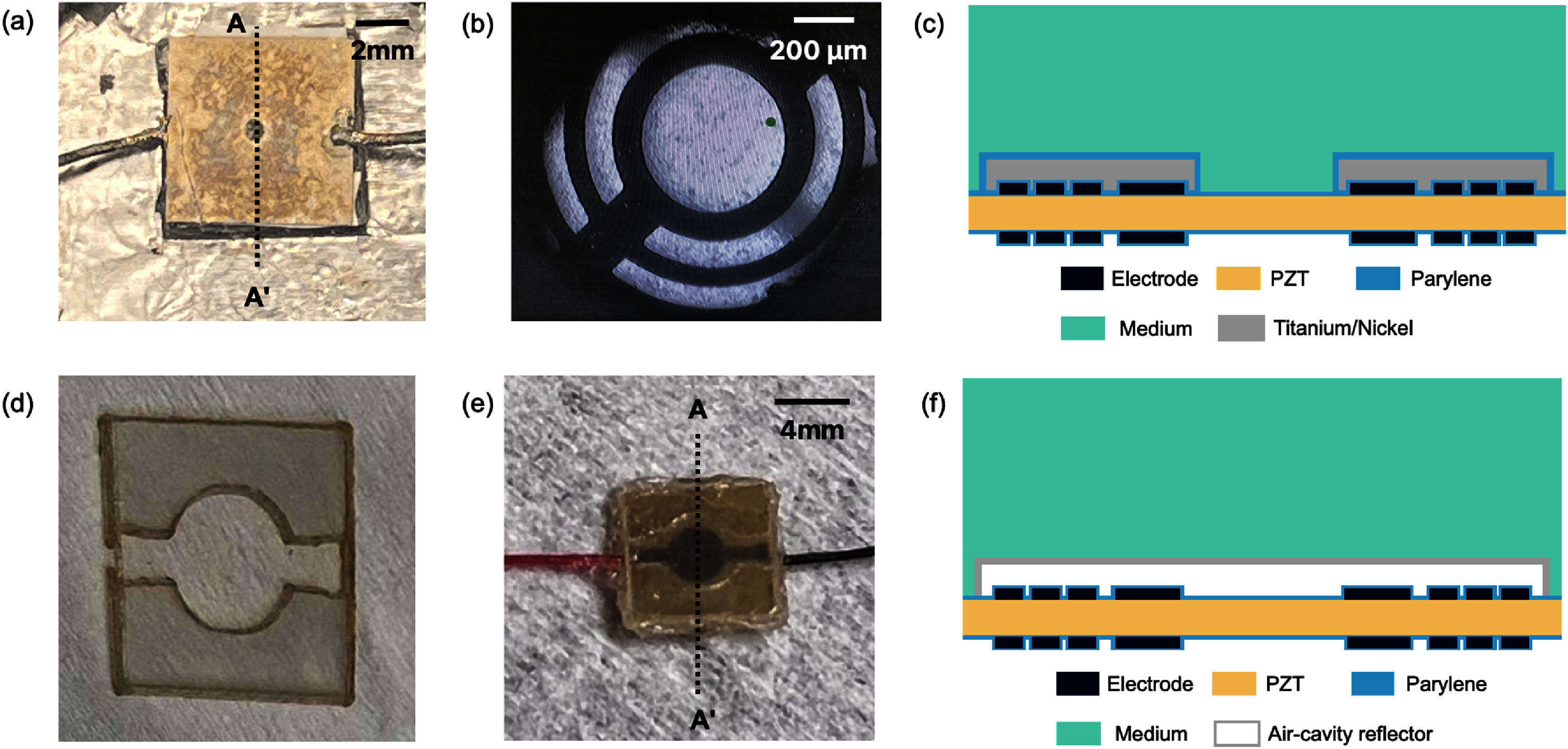
Photos of (a) the fabricated low-EMI SFAT and (b) the microscopic image validating the translucency of low-EMI SFAT. (c) The cross-sectional diagram ((across the AA′ dashed line in (a))) of the low-EMI SFAT. Photos of (d) an air-cavity reflector made from three pieces of laser-machined acrylic sheets and (e) a FUS-blocking control SFAT with the air-cavity reflector attached, and insulated through Parylene coating. (f) The cross-sectional diagram ((across the AA′ dashed line in (e))) of the FUS-blocking control SFAT with an air-cavity reflector.

Nickel is selected as the EMI shielding metal for its low skin depth due to its high permeability. A 1.25 *µ*m thick Nickel layer is deposited on top of the SFAT with 100 nm thick Titanium (Ti) layer acting as an adhesion layer. Before the deposition, a thermal release tape (Semiconductor Equipment Crop.), shaped in a circle with a 1.5 mm diameter, is manually placed at the center of SFAT (figures 3(a) and (b)). This serves as a mask to protect the central area in order to preserve the translucency of SFAT, when the thermal release tape is released by elevating the temperature to 90 °C in the oven.

### 2.4. FUS-blocking control SFAT

As an SFAT also gets heated due to dielectric and I^2^R heating (during patch-clamp experiments) which is a potential modulatory source of neuronal activity, a FUS-blocking control device is designed and fabricated with identical ring patterns (15 rings and a 400 *µ*m focal length) and operating frequency (18.4 MHz). To block the acoustic wave for a FUS-blocking control device, an air-cavity reflector (made with laser machining and composed of three stacked acrylic sheets, each 127 *µ*m thick) is added to the top of SFAT. The air-cavity reflector uses the high reflection coefficient (99%) between air and any solid material, due to the large acoustic impedance difference between air (0.4MRayl) and solid (over 1MRayl) [43], so that no acoustic wave may pass through it. The combined thickness of the acrylic sheets results in an air-cavity with about 390 *µ*m air gap (figure 3(d)). These acrylic sheets are attached to the SFAT with super glue and sealed with an additional layer of 20 *μ*m thick Parylene (figure 3(e)).

## 3. Experiment design

### 3.1. Whole-cell patch clamp experiment design

**Figure 4.**
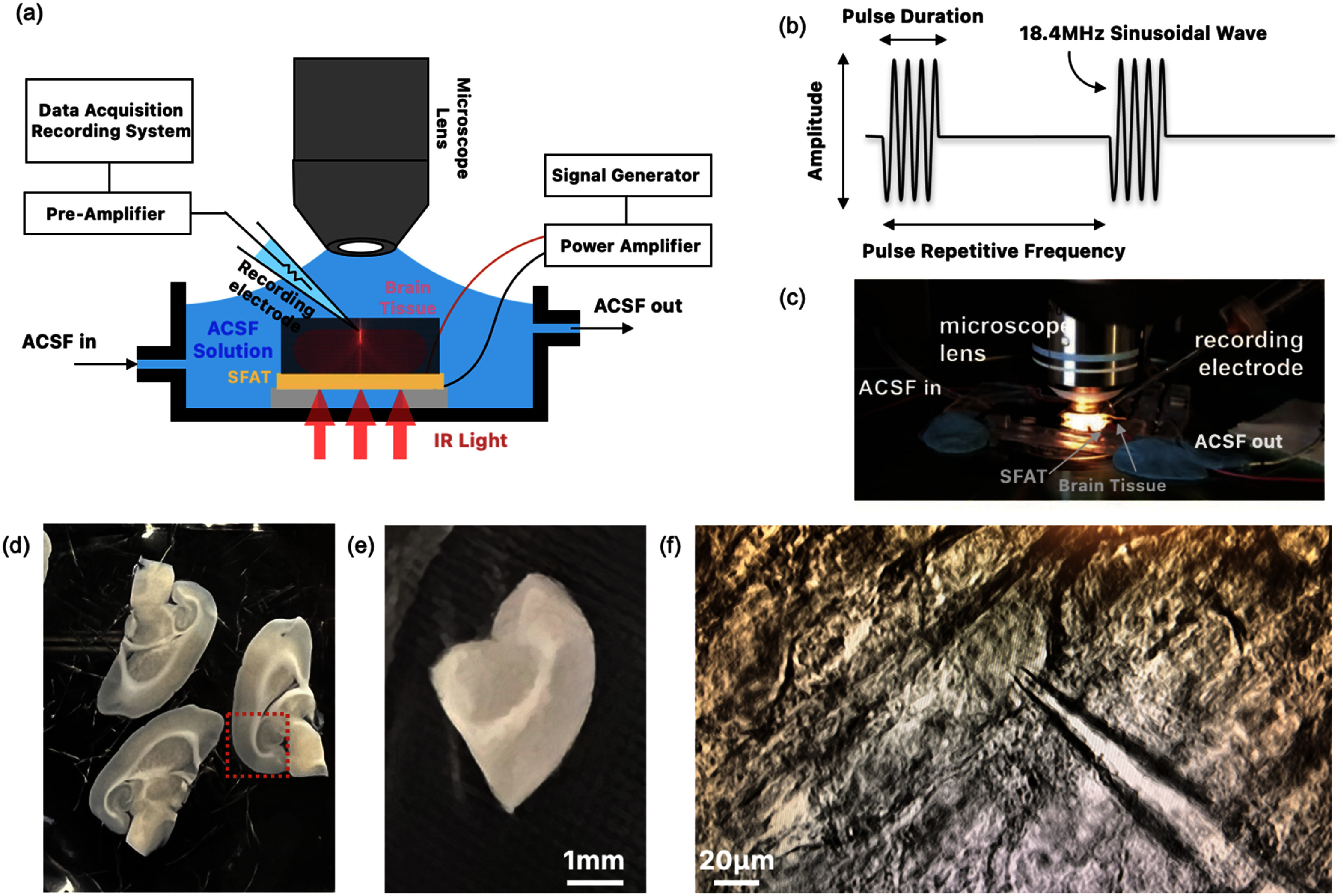
(a) Conceptual diagram of the patch clamp experimental setup, overlaying the focused ultrasound simulation on the tissue diagram and (b) pulsed operation of SFAT with the definition of the number of cycles, pulse duration, pulse repetitive frequency (PRF), and operating frequency. Photos of (a) the whole-cell patch clamp experiment setup with an SFAT, (d) acute slices from 3–4 weeks old Sprague Dawley rat, (e) hippocampus (red box in (d)) region of the slice, and (f) a photo of the patched neuron with the micropipette on the hippocampus tissue over the SFAT through which the light passes through and illuminates the tissue.

The whole-cell patch clamp technique allows recording of the electrical activity of individual neurons (or cells) [44], and is carried out with FUS produced by an SFAT in a setup illustrated in figure 4.

An SFAT is placed at the bottom of a transparent chamber with a continuous flow of artificial cerebrospinal fluid (ACSF) at a rate of 2–3 ml min^−1^, while the rat brain slice is positioned on top of the SFAT (figure 4(c)), with the light illumination from under the SFAT. The hippocampal brain slices (350–400 *µ*m in thickness, approximately 5 × 5 mm^2^ in size) are prepared from Sprague Dawley rat (without sex-specific selection, 3–4 weeks old, and 250–300 g body weight) and transferred into a pre-chamber filled with ACSF (aerated with 95% O_2_/5% CO_2_) composed of 124 NaCl, 2.5 KCl, 1.25 KH_2_PO_4_, 1 MgSO_4_, 2 CaCl_2_, 26 NaHCO_3_, and 11 D-glucose, with all the numbers in mM. Then the slices are incubated in ACSF at 35 °C for the first 15 min and kept in ACSF at room temperature for at least 1 h before recording the neuronal activity.

For a whole-cell patch clamp, a borosilicate glass capillary tube is pulled with a horizontal puller (P-1000, Sutter Instrument) to create a micropipette. Inside the micropipette, the patch pipette solution is filled with 125 K-gluconate, 10 KCl, 10 HEPES, 1 EGTA, 2 MgCl_2_, 0.1 CaCl_2_, and 4 ATP-Na_2_. During experiments, to record the neuronal activities, the micropipette tip is attached to a neuron in the CA1 region of hippocampus. The recording electrode inside the micropipette is connected to the preamplifier and the recording acquisition system (MultiClamp 700B Microelectrode Amplifier from Molecular Devices) with a sampling rate of 10 kHz. The SFAT is driven with pulsed sinusoidal signals generated by a function generator (AFG-3252, Tektronix Inc.) and amplified by a power amplifier (75A250, Amplifier Research Corp.).

### 3.2. FUS stimulation parameters design

An SFAT delivers a series of pulsed acoustic waves to the target neuron located at the focal length with precise control over the timing and reduced heat generation, compared to continuous acoustic stimulation. The first key parameter is the peak-to-peak voltage, which is related to the acoustic intensity. For this, three values are chosen for the low, mid, and high voltage ranges: 40, 60, and 120*V*_pp_, with corresponding *I*_SAPA_ values being 0.44, 2.11, and 8.44 W cm^−2^, respectively.

The other two key parameters are the PRF and the number of cycles per pulse. During transducer operation, a series of acoustic pulses can be characterized by the PRF, as illustrated in figure 4(b), along with the number of cycles per pulse which depends on the pulse duration (PD) as well as the period (*T*_s_) and frequency (*f*_s_) of the sinusoidal waves:

\begin{equation*}{\text{Number of cycles}} = \,{\text{PD}}/\,{T_s}\, = \,{\text{PD}}\, \times {f_s}.\end{equation*}

In our experiments, various PRFs are tested, ranging from 1 Hz to 200 Hz, with the number of cycles per pulse varying between 5 k and 65 k cycles per pulse. Using equation (4), *I*_SPTA_ values are computed to span from 1 to 1031 mW cm^−2^. The ultrasound stimulation parameters are mostly within the FDA safety guidelines for ultrasound in biomedical application (*I*_SPPA_ <190 W cm^−2^ and *I*_SPTA_ <720 mW cm^−2^) [45], although current FDA approval applies mostly to ablative procedures like pallidotomy, not neuromodulation. A higher peak-to-peak voltage results in more heat generation from SFAT, posing a risk of damage to either the transducer or the nearby tissue. The limit of the driving voltage is also influenced by the number of cycles per pulse and PRF, as mechanical stress and heat on and from SFAT accumulate over the pulse duration. Table 2 presents examples of key ultrasound parameters used in the patch-clamp experiments. These experiments, with systematically varied parameters, are meticulously designed to assess the effects of different *I*_SAPA,_ *I*_SATA,_ PD, and PRF. For example, the first three parameter sweeps in table 2 evaluate the impact of PD while maintaining a constant PRF. In particular, the number of cycles per pulse is varied and selected among 5000, 10 000, 15 000, 25 000, 50 000, and 65 000 with a fixed I_SAPA_ of 0.94 W cm^−2^ and PRF of 20 Hz. On the other hand, the last three rows are designed to examine the effect of PRF while keeping the PD consistent. Similarly, FUS stimulation with an *I*_SAPA_ of 8.44 W cm^−2^ and a fixed PD of 45 000 cycles per pulse varies its PRF across 5, 10, 15, 20, 25, and 50 Hz. Distinct experimental conditions include variations in ultrasound parameters, cell identity, and device type (active or FUS-blocking device).

**Table 2.** Examples of ultrasound parameters used in the FUS patch clamp experiment.

Peak to peak voltage amplitude (*V*_pp_)	Spatial average Pulsed average acoustic intensity, ${I_{{\text{SAPA}}}}$ (*W* cm^−2^)	Spatial average Pulsed average acoustic intensity, ${I_{{\text{SAPA}}}}$ (kPa)	Pulse repetitive frequency (Hz)	Number of cycles per pulse	Duty cycle (%)	Spatial average time average acoustic intensity, ${I_{{\text{SATA}}}}$ (mW cm^−2^)
40	0.94	160	20	5000–65 000	0.54–7.07	5.09–66.22
60	2.11	262	20	5000–65 000	0.54–7.07	13.45–174.86
120	8.44	524	20	5000 −65 000	0.54–7.07	42.10–547.36
40	0.94	160	330	30 000	50.80	504.30
60	2.11	262	120	30 000	19.56	499.25
120	8.44	524	40	30 000	6.52	505.25
40	0.94	160	10–200	45 000	2.45–48.91	22.92–458.45
60	2.11	262	5–200	45 000	1.22–48.91	25.78–1031.52
120	8.44	524	5–50	45 000	1.22–12.23	94.74–947.35

The FUS stimulation with varying parameters is applied to the patched neuron for 30 s, followed by a 2 min resting period that allows cells to recover from any potential thermal effects induced by the ultrasound. Each patched cell typically receives multiple ultrasound stimulations until patch integrity is lost or the cell is damaged. The experiments have been systematically conducted with varied ultrasound parameters on the different cells and different devices, as shown in table 2, culminating in a comprehensive dataset across 323 distinct experimental configurations. Out of a total of 323 experiments, FUS is delivered with the active devices in 281 experiments, while the FUS-blocking control devices are used in the remaining 42 experiments. Patch clamp recordings are performed on 78 distinct tissue slices obtained from 29 animals (both male and female). A total of 39 different FUS pulse parameter sets are applied, while multiple FUS stimulations are delivered to the same neuron until the patch seal is lost, resulting in some neurons receiving multiple stimuli and others only a single stimulus. The maximum number of FUS stimuli applied to a single neuron is 15, with a resting period of 2 min between stimuli.

In some cases, a small positive or negative current is injected into the cell to adjust the baseline membrane potential. For example, when assessing the effect of FUS on neurons with a low resting membrane potential (without spontaneous APs), a positive current is injected to depolarize the baseline membrane potential, allowing a clearer comparison of the number of APs before, during, and after FUS stimulation. Conversely, in neurons with a high baseline membrane potential that shows bursting spikes, applying a negative current inhibits the bursting spikes and stabilizes the neuronal firing pattern, facilitating analysis of the FUS effect. The amount of the injected current is maintained to be no more than ±40 pA to prevent the neuron from responding to the electrical stimulation.

## 4. Experiment result

### 4.1. Neuromodulation effect of FUS

**Figure 5.**
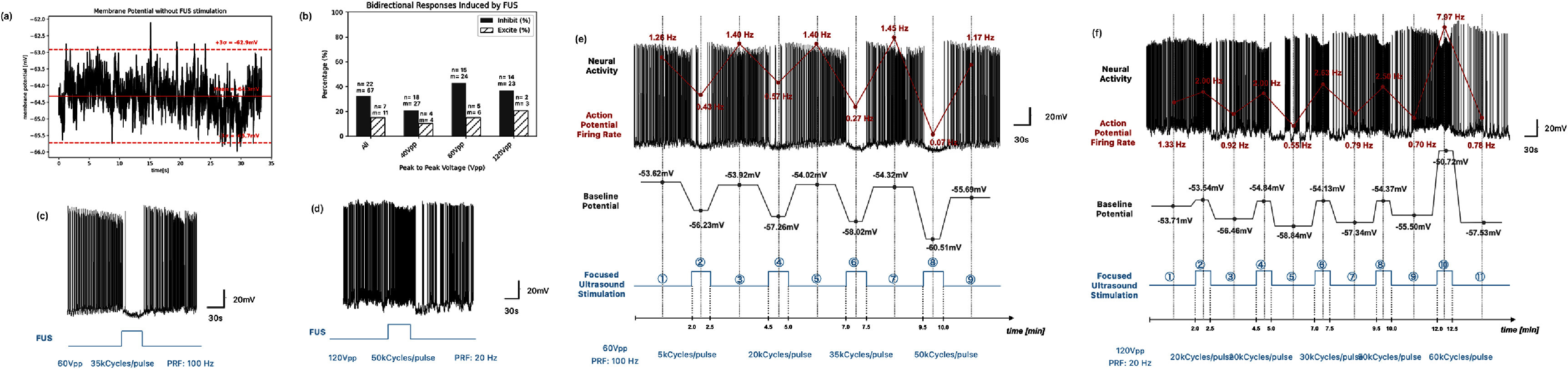
(a) The membrane potential with the absence of focused ultrasound stimulation, showing the mean and ±2% or ±3 sigma(*σ*) range (b) the inhibition and excitation rates (in percentage) induced by FUS across the different voltage levels applied to the SFAT, regardless of the FUS pulsing parameters such as PRF and pulse duration. *n* and m represent the number of animals and the tissues used in the patch clamp experiments, respectively. (c)–(f) The recorded action potentials (black) along with the activation of focused ultrasound stimulation (blue). (c) A sample inhibition effect of the FUS with the parameters set at 60*V*_pp_ (*I*_SAPA_ = 2.11 W cm^−2^), 35 kCycles/pulse (PD = 1.90 ms) and a PRF of 100 Hz (which results in 60% yield of successful inhibition). (d) A sample excitation effect of the FUS with the parameters set at 120*V*_pp_ (*I*_SAPA_ = 8.44 W cm^−2^), 50 kCycles/pulse (PD = 2.72 ms) and a varied PRF of 20 Hz (which results in 60% yield of successful excitation). Series of (e) the inhibition and (f) the excitation effects with 30 s of FUS, followed by 2 min of resting period.

Though evidence of the inhibitory effect by FUS neural stimulation has been more prevalent [9, 25], recent studies indicate that FUS can modulate the firing rate of the APs in both directions when its PRF and the pulse duration are tuned [28–31]. We have carried out a number of experiments with various PRFs and cycle numbers per pulse to find the optimal parameters that inhibit or excite the neuronal activities. The classification of neuronal activity as either inhibition or excitation is based on changes in the baseline membrane potential. Specifically, a depolarization exceeding 2% during FUS stimulation is considered indicative of excitation, while hyperpolarization greater than 2% signifies inhibition. Experiments with baseline membrane potential changes within ±2% are classified as ‘no response’ to FUS. The criterion is determined by recording the membrane potential in the absence of FUS stimulation for 30 s (figure 5(a)) and subsequently calculating the mean and standard deviation (*σ*). A threshold of ±2% relative to the mean is set, corresponding to approximately 3*σ*, which encompasses 99.7% of the data under the assumption that neuronal activity without stimulation follows a normal distribution, shown in figure 5(a).

Overall, 30% of cells exhibit inhibition in response to FUS, 15% display excitation, and 55% show no response, regardless of the FUS parameter, tissue, and animal subjects. Table 3 provides a summary of the number and percentage of inhibitory and excitatory responses observed for each device, along with the number of tissues (*m*) and the animals (*n*) used.

**Table 3.** Summary of neuronal inhibition and excitation events and rates induced by SFATs with all different FUS parameters onto all patched neurons.

Device type	Inhibition	Excitation	No response	Total events	Number of tissues (*m*)	Number of animals (*n*)
Total	99 (30%)	48 (15%)	176 (55%)	323	78	29
Active device	92 (33%)	42 (15%)	147 (52%)	281	67	22
FUS-blocking control device	7 (16%)	6 (14%)	29 (70%)	42	11	7

Across all voltage levels, the inhibition effect is at least twice as prevalent as the excitation effect. Figure 5(b) presents the breakdown of inhibition and excitation rates across different applied voltage levels. These rates are calculated by counting the number of events, independent of animal, tissue, or other pulsed parameters such as PRF and pulse duration. Notably, the inhibition effect with 60*V*_pp_ FUS demonstrates the most pronounced response. Specifically, the inhibition rate is approximately 43% (39 out of 91 events, *n* = 15, *m* = 24), which is three times higher than the excitation rate of about 14% (10 out of 91 events, *n* = 15, *m* = 24) (figure 5(b)). The baseline membrane potential change of 2% results in changes in the AP firing rate. The inhibition effect, which suppresses the firing rate either partially or completely, is observed most dominantly with the ultrasound parameters set at the 60*V*_pp_ (*I*_SAPA_ = 2.11 W cm^−2^), 35 000 cycles/pulse (PD = 1.90 ms) and a PRF of 100 Hz, with corresponding *I*_SATA_ values of 130 mW cm^−2^. The AP is completely suppressed during the ultrasound stimulation (figure 5(c)). This operating parameter yields a 60% success rate in the inhibition effect, with 10 out of 17 cases showing effectiveness, while raising the cell temperature by less than 1 °C. The significantly lower inhibition rate observed with the FUS-blocking control device suggests that the inhibition effect is closely associated with FUS. Additionally, the inhibition effect is consistently observed with repeated ultrasound stimulation at two-minute intervals, using varying FUS parameters. Figure 5(e) shows consecutive FUS applications on the same cell, with four different FUS conditions varying the number of cycles per pulse from 5 k, 20 k, 35 k and 50 k, while keeping *I*_SAPA_ and PRF fixed at 2.11 W cm^−2^ and 100 Hz, respectively. The plot also clearly demonstrates reductions in both the AP firing rate and the baseline membrane potential induced by the FUS stimulation.

In contrast, the excitation effect (figure 5(d)) is observed less frequently and occurs at a similar rate (∼15%) in both the active and FUS-blocking SFAT devices, indicating no significant difference between the two. Notably, excitation was observed under FUS parameters associated with greater temperature increases. For example, using a parameter set at 120*V*_pp_ (*I*_SAPA_ = 8.44 W cm^−2^), 50 000 cycles/pulse (PD = 2.72 ms) and a PRF of 20 Hz—corresponding *I*_SATA_ values of 458 mW cm^−2^ and temperature rise of 3.5 °C (including the conduction heat)—excitation was detected in 60% of trials. Similar to the inhibitory response, the excitation effect is also consistently observed with successive FUS stimulations at higher ultrasound intensities (figure 5(f)), although recovery times for the cells take longer due to the higher temperature increase. In experiments involving consecutive FUS applications on the same cell with pulse durations ranging from 20 k to 65 k cycles per pulse (i.e. 20 k, 35 k, 50 k, and 65 k) at an *I*_SAPA_ of 8.44 W cm^−2^ and a PRF of 20 Hz, baseline membrane potentials are gradually depolarized, resulting in increased firing rates. Table 4 summarizes the FUS parameters used in figures 5(e) and (f), along with the corresponding electrophysiological responses.

**Table 4.** Focused ultrasound stimulation parameters and induced electrophysiological data.

	Inhibitory response (figure 5(e))								Excitatory response (figure 5(f))						
	Spatial peak pulsed average acoustic intensity, ${I_{{\text{SPPA}}}}$ (W cm^−2^)	Pulse repetitive frequency (Hz)	Number of cycles per pulse	Duty cycle (%)	Spatial average time average acoustic intensity, ${I_{{\text{SATA}}}}$ (mW cm^−2^)	Baseline membrane potential (mV)	Action potential firing rate (Hz)		Spatial peak pulsed average acoustic intensity, ${I_{{\text{SPPA}}}}$ (W cm^−2^)	Pulse repetitive frequency (Hz)	Number of cycles per pulse	Duty cycle (%)	Spatial average time average acoustic intensity, ${I_{{\text{SATA}}}}$ (mW cm^−2^)	Baseline membrane potential (mV)	Action potential firing rate (Hz)
①	Rest: no FUS stimulation					−53.62	1.26	①	Rest: no FUS stimulation					−53.71	1.33
②	2.11	100	5000	2.71	57.31	−56.23	0.43	②	8.44	20	20 000	2.17	183.38	−53.54	2.00
③	Rest: no FUS stimulation					−53.92	1.40	③	Rest: no FUS stimulation					−56.46	0.92
④	2.11	100	20 000	10.87	229.23	−57.26	0.57	④	8.44	20	20 000	2.17	183.38	−54.84	2.00
⑤	Rest: no FUS stimulation					−54.02	1.40	⑤	Rest: no FUS stimulation					−58.84	0.55
⑥	2.11	100	35 000	19.02	401.15	−58.02	0.27	⑥	8.44	20	35 000	3.80	320.92	−54.13	2.63
⑦	Rest: no FUS stimulation					−54.32	1.45	⑦	Rest: no FUS stimulation					−57.34	0.79
⑧	2.11	100	50 000	27.17	573.07	−60.51	0.07	⑧	8.44	20	50 000	5.44	458.85	−54.37	2.50
⑨	Rest: no FUS stimulation					−55.69	1.17	⑨	Rest: no FUS stimulation					−55.50	0.70
								⑩	8.44	20	65 000	7.07	595.99	−50.72	7.97
								⑪	Rest: no FUS stimulation					−57.53	0.78

### 4.2. Effect of PRF and pulse duration on inhibition

The rate of the inhibition effect is influenced by FUS parameters such as PRF and the number of cycles per pulse (or pulse duration), with these two parameters collectively determining the pulse duty cycle. Further analyses are conducted to examine the dependency of the FUS inhibition effect on PRF and pulse duration as follows.

**Figure 6.**
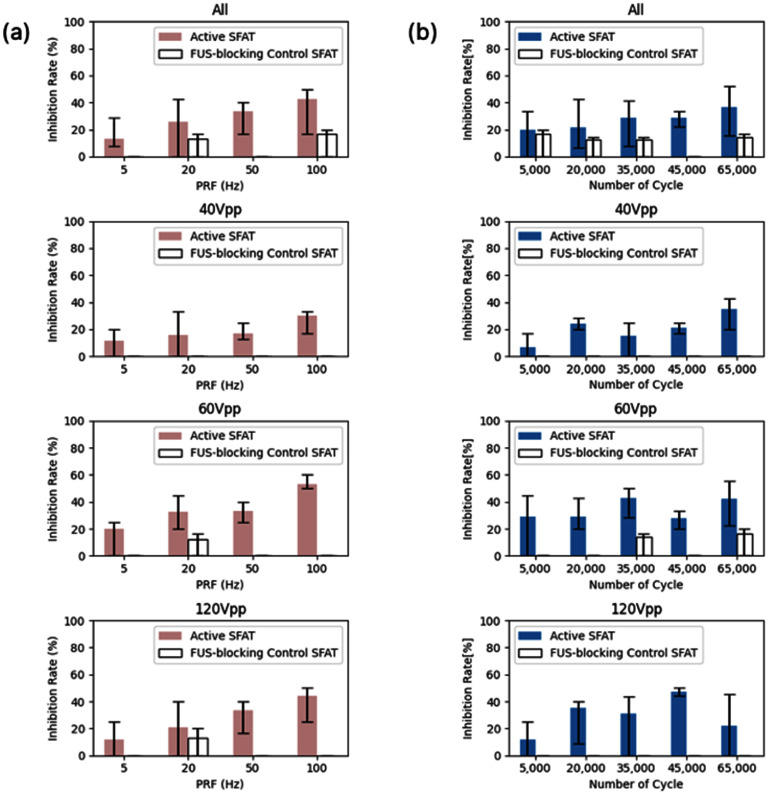
The inhibition rates (a) as a function of the PRF (5, 20, 50, and 100 Hz), averaged over different numbers of cycles per pulse (5 k–65 k) and (b) as a function of the number of cycles per pulse (5 k–65 k), averaged across different PRF values (5, 20, 50, and 100 Hz), at different applied voltage levels (all, 40 *V*_pp_, 60 *V*_pp_, and 120 *V*_pp_, from the top to bottom).

To investigate PRF dependency, the inhibition rate is calculated with the different PRF values of 5, 20, 50, and 100 Hz, while the number of cycles per pulse fixed at 5 k, 20 k, 35 k, 45 k, and 65 k, corresponding to the pulse duration of 0.27, 1.09, 1.90, 2.45, and 3.53 ms, respectively. For each PRF, the inhibition rate is averaged across all tested numbers of cycles, and the average rates, along with their minimum and maximum ranges, are plotted in figure 6(a). This analysis is performed at three different voltage levels (40, 60, and 120*V*_pp_ or *I*_SAPA_ = 0.94, 2.11, 8.44 W cm^−2^) with the results shown in separate panels in figure 6(a). The top panel presents the overall average inhibition rate across all voltage levels. For example, the first bar in the top plot represents the average inhibition rate at PRF = 5 Hz, where the number of cycles is swept from 5 k to 65 k across all voltage conditions. At PRF = 5 Hz, the averaged inhibition rate across all pulse durations was 13.16% (10 inhibited cases out of 76 total trials), while the maximum inhibition rate of 28.57% (2 out of 7) and the minimum rate of 7.69% (1 out of 13) were observed at 45 k and 35 k cycles, respectively. These maximum and minimum values are represented by the error bars in each bar plot.

The same methodology is applied to the FUS-blocking control device, and its results are included for comparison. Across all voltage levels, the inhibition rate increases with higher PRF values, reaching a maximum of 60% at a FUS parameter of 60*V*_pp_ (*I*_SAPA_ = 2.11 W cm^−2^), 35 k cycles per pulse, and a PRF of 100 Hz. These findings suggest the strong dependency of the inhibition effect on PRF. Specifically, a higher PRF increases the likelihood of inhibiting AP from the pyramidal cells in the hippocampal CA1 region.

Similarly, the dependency on pulse duration is analyzed with PRF fixed at 5, 20, 50, and 100 Hz, as the number of cycles per pulse is varied across 5 k, 20 k, 35 k, 45 k, and 65 k. The average inhibition rates for each pulse duration (determined by the number of cycles per pulse) are plotted in figure 6(b), along with their corresponding minimum and maximum values. Unlike the PRF dependency, pulse duration does not significantly influence the inhibition rate. Across all voltage levels, the inhibition rate consistently falls within the range of approximately 20%–40%, regardless of the number of cycles. These findings suggest that while PRF plays a critical role in modulating the inhibition effect, pulse duration has a relatively limited impact.

However, it is important to note that the inhibition rate is calculated by aggregating data across a range of pulse durations or PRF values under each tested condition. This averaging approach is necessary due to the limited number of experimental replicates available for each unique parameter combination. As a result, the inhibition rates shown in figure 6 represent pooled outcomes rather than independent, fully balanced measurements for each parameter. While this analysis effectively reveals overall trends, it may mask potential interactions or finer dependencies between PRF and pulse duration. Future studies with expanded sample sizes and systematically varied experimental conditions will be valuable to further delineate the individual and combined contributions of these ultrasound parameters to the inhibition effect.

### 4.3. Thermal effect

Acoustic waves used in neurostimulation may result in heat generation in the tissues. A previous study suggests that heat is the primary effector of ultrasound neuromodulation, claiming that FUS neural modulation may be observed only with a pulse duration long enough to generate heat exceeding 2 °C [26]. However, a recent study in normal intact mice indicates that acoustic energy stimulates neuronal activity without a significant rise in brain temperature (<0.01 °C) [23]. To verify the thermal effects of the FUS, it is crucial to control and measure temperature changes in the neurons during stimulation.

**Figure 7.**
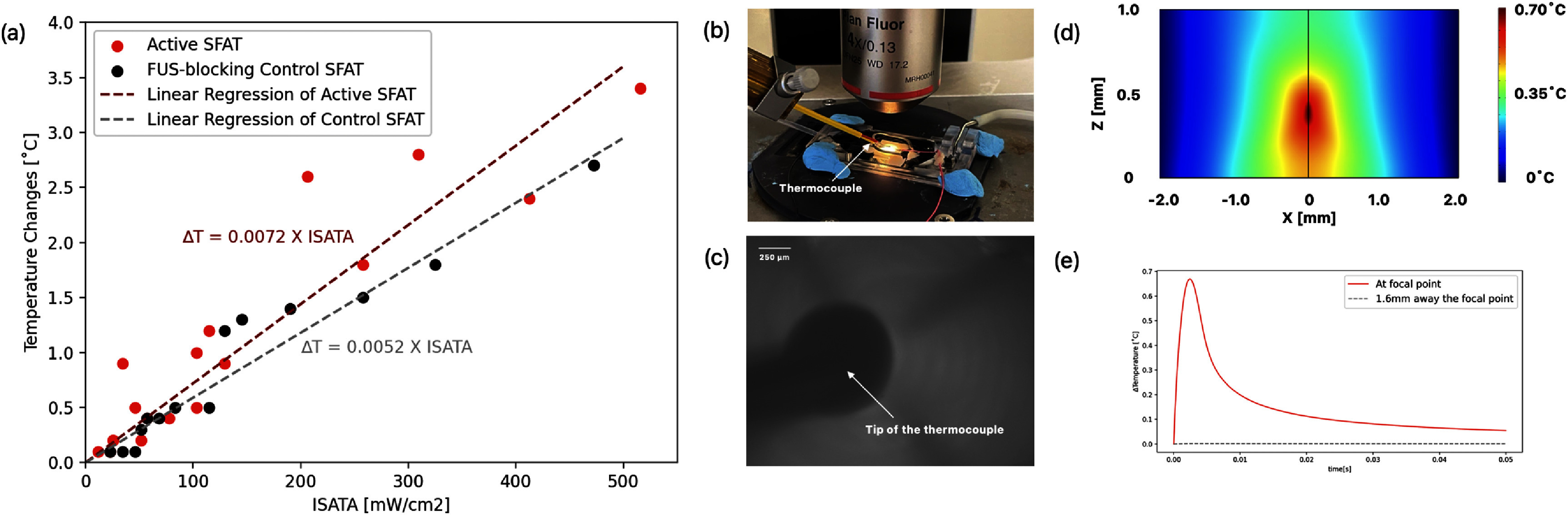
(a) Measured temperature rise on the surface of the brain tissue with the activation of the active SFAT and the FUS-blocking control SFAT. (b) Photo of the experimental setup to measure the temperature changes with a thermocouple placed on top of the tissue and aligned to the center of the SFAT. (c) Microscopic photo showing the tip of the thermocouple on a brain slice above an SFAT. (d) Simulated temperature profile at the acoustic focal point, showing a maximum temperature rise of 0.67 °C under the following FUS parameters: 120 *V*_pp_, 50 000 cycles per pulse (2.7 ms pulse duration), and a PRF of 20 Hz. (e) Temperature changes at the focal point (red line) and an off-focus location (blue line) over a single PRF cycle (50 ms), indicating a localized heating of less than 0.67 °C and full thermal recovery within one cycle.

To examine the thermal effects induced by FUS, temperature changes on the top surface of a brain slice sitting on top of an SFAT are measured without patching the cell, using a miniature k-type thermocouple with an 800 *µ*m diameter tip (HH506RA, Omega Engineering Inc.). With both the active SFAT and FUS-blocking control SFAT, various driving parameters with *I*_SPTA_ ranging from 5 to 500 mW cm^−2^ are tested, and the temperature rise on the tissue surface is measured to increase linearly as the *I*_SATA_ of FUS is increased (figure 7(a)). The temperature rise after a 30 s actuation of the transducer with an active SFAT is 0.3 °C–3.4 °C, while it is 0.2 °C–2.7 °C for the FUS-blocking control SFAT, depending on the *I*_SATA_. The difference between the two cases is not supposed to be due to the difference in FUS delivered to the tissue, since the FUS intensity level is not high enough to produce such a difference. Rather, the FUS-blocking control SFAT with an air cavity covering the whole SFAT surface blocks not only the generated acoustic waves, but also heat conduction from the surface of the transducer heated due to I^2^R heating on the patterned electrodes. Even if the difference is all due to FUS, the measurements indicate that temperature rise due to the FUS alone would certainly be less than 1 °C up to *I*_SATA_ of 500 mW cm^−2^.

Although thermocouple measurements provide a rough estimate, they are subject to several limitations such as acoustic absorption by the probe, potential interference with the ultrasound field, and limited spatial resolution. Therefore, we supplement the measurements with thermal simulations using COMSOL. In the simulation, we model a representative FUS condition: 120*V*_pp_, 50 000 cycles (2.7 ms pulse width) per pulse, and a PRF of 20 Hz, which gives the highest chance of excitatory modulation effect. To approximate the spatial peak intensity (*I*_SPPA_) required for the simulation, we scale the measured *I*_SAPA_ by the area ratio of the hydrophone diameter (200 *µ*m) to the estimated focal spot size (∼46 *µ*m), i.e. (200 *µ*m/46 *µ*m)^2^, assuming a Gaussian-like field distribution. Though this estimation introduces uncertainty due to the spatial averaging effect of the hydrophone and idealized thermal boundary condition in the model, the simulation provides a reasonable approximation. When the simulation is run over one PRF cycle (50 ms), the results show that the maximum temperature rise at the acoustic focal point (*r, z*) = (0,0.4 mm) is 0.67  °C (figure 7(d)), while at an off-focus location, (*r, z*) = (1.6 mm,0.4 mm) the temperature increase is almost zero. Importantly, the temperature returns to baseline within a single PRF period (figure 7(e)). These results confirm that the temperature rise induced by FUS under the tested parameters remains well below 1 °C, supporting the conclusion that thermal effects are minimal.

## 5. Discussion

Our study demonstrates that FUS stimulation using a MEMS-based SFAT can induce bidirectional neuromodulatory effects on CA1 pyramidal neurons in hippocampal brain slices. Through patch-clamp experiments, we observe that FUS led to neuronal inhibition in 30% and excitation in 15% of cells. In contrast, control experiments using a FUS-blocking SFAT produced both inhibition and excitation in approximately 15% of cells, with the majority (70%) showing no response. This contrast suggests that mechanical energy from the FUS contributes to the inhibitory effects, while the excitatory effects may be primarily due to thermal influences. Interestingly, the excitatory response did not significantly differ between the active and heat-only conditions, prompting us to interpret excitation effects more cautiously. We hypothesize that these excitatory responses may result from heat generated by the transducer, either via conductive transfer through the tissue or via ultrasound-induced heat. Thermal simulations confirm that under stimulation conditions corresponding to an I_SATA_ of 458 mW cm^−2^, the local temperature rise remains below 0.67 °C, indicating minimal thermal accumulation at the focal region (figure 7(d)). However, heat from transducer operation can elevate the temperature by up to 3.4 °C, potentially enough to depolarize the membrane and trigger excitatory activity (figure 7(a)). This thermal influence also provides an explanation for the reduced inhibition rate observed at 120*V*_pp_ (*I*_SAPA_ = 8.44 W cm^−2^), compared to 60*V*_pp_ (*I*_SAPA_ = 2.11 W cm^−2^) (figure 5(a)). Despite the higher acoustic intensity of 8.44 W cm^−2^ the accompanying increase in temperature appears to counteract the inhibitory effect, underscoring the complex interplay between thermal and mechanical mechanisms in ultrasound neuromodulation.

Based on the observation and findings from the patch clamp experiments, we hypothesize that FUS can influence neurons through two primary mechanisms: mechanical stimulation caused by ultrasound waves and thermal stimulation generated by heat. Thermosensitive ion channels are known to modulate neuronal excitability through their varying activating temperature and temperature gradient [46]. Bachtold *et al* demonstrated that continuous-wave ultrasound induced neuronal inhibition in hippocampal slices, attributing the effect to thermal mechanisms that reduce excitability [47]. Given that excitatory thermosensitive ion channels outnumber their inhibitory counterparts, it is plausible that the excitatory effects observed in our study are primarily mediated by temperature increases generated by the transducer and the ultrasound focus. Among thermosensitive channels, members of the transient receptor potential (TRP) family have been extensively investigated in the context of FUS neuromodulation. For instance, Yoo *et al* reported that FUS can activate TRP channels, leading to elevated intracellular Ca^2+^ levels and subsequent activation of Ca^2+^-gated Na^+^ channels, resulting in membrane depolarization and neuronal excitation [48]. Table 5 summarizes recent studies examining the involvement of TRP channels in FUS neuromodulation and their proposed mechanisms of action.

**Table 5.** Focused ultrasound neuromodulation studies related to TRP channels.

Subfamily	Subtype	Primary mechanism	Previous study
TRPC	TPRC1	G-protein coupled receptor (GPCR)/ mechanical stimuli	Yoo *et al*^a^ [48]
	TRPC1		Burk *et al*^a,c^ [49]
TRPV	TRPV1	Temperature (Heat)	Yoo *et al*^b^ [48]
	TRPV2	Heat/ membrane stretch	
	TRPV4	Heat/ membrane stretch	
TRPM	TRPM2	Intracellular calcium	Yang *et al*^a^ [50]
	TRPM7	Intracellular calcium	Yoo *et al*^b^ [48]
TRPA	TRPA1	Temperature (Cold) and mechanical stimuli	Oh *et al*^a^ [32]
TRPP	TRPP1/TRPP2	Mechanical stimuli	Yoo *et al*^a^ [48]

^a^Excitatory effect of FUS Neurostimulation.

^b^No significant effect of FUS Neurostimulation.

^c^Not cortical neurons.

In addition to TRP channels, K2P channels are known for their responsiveness to various stimuli, including heat, chemical, and mechanical factors. They play a role in facilitating outward K+ ion movement, resulting in hyperpolarization of the membrane potential and subsequent neuron inhibition [51]. The hypothesis is that K2P ion channels are responsible for that the inhibitory effect of FUS, as K2P ion channels encompass various subfamilies, including TWIK, TREK, TASK, TRESK, and others. Sorum *et al* suggests the TRAAK channel, which is a subtype of TRESK channel, as the primary inhibitory mechanism of FUS neuromodulation [52]. Among these subfamilies, the TRESK channel emerges as a particularly promising candidate potentially associated with FUS neuromodulation, as its activation mechanism is modulated by intracellular calcium concentration [53], an ion reported to increase with FUS [48].

Finally, it is worth noting that Suarez-Castellanos *et al* (2021) reports that a single pulse of FUS can evoke local field potentials in hippocampal slices [54], an excitatory response not observed in our study. This discrepancy may be attributed to differences in experimental setup, stimulus waveform, or acoustic parameters, highlighting the importance of these factors in determining the neuromodulatory outcomes of FUS.

## 6. Summary

A FUS generated by a SFAT has been systematically used in extensive whole-cell patch-clamp experiments to investigate its neuromodulation effects on the central nervous system, particularly on the CA1 region of acute hippocampal slices from juvenile (P14–P21) Sprague-Dawley rats of both sexes. The SFAT is specially designed and fabricated on a 127 *µ*m thick PZT with annular electrodes (and no electrode on the circular center area for IR light to pass through for good visibility of the cells on the tissue). This SFAT operating at 18.4 MHz has a small focal diameter of 46 *µ*m, offering fine spatial resolution during the patch-clamp study.

The FUS parameter at 60*V*_pp_ (*I*_SAPA_ = 2.11 W cm^−2^), 35 kCycles/pulse (pulse duration = 1.90 ms) and PRF of 100 Hz yields 60% success rate of the inhibition effect, while operating SFAT at 120*V*_pp_ (*I*_SAPA_ = 8.44 W cm^−2^), 50 kCycles/pulse (pulse duration = 2.72 ms) and a PRF of 20 Hz excites the neuron with a 60% success rate. Temperature measurements indicate a linear relationship between applied acoustic energy and local heating, with maximum temperature rises of up to 3.4 °C under excitation conditions and 2.7 °C with a control device that blocks acoustic transmission. These findings suggest that thermal effects, particularly the activation of thermosensitive ion channels such as TRP and K2P, play a role in mediating the observed excitatory and inhibitory outcomes.

Furthermore, systematic variation of stimulation parameters revealed that the inhibitory effect is strongly dependent on PRF, but relatively insensitive to pulse duration within the tested range. These results underscore the importance of both acoustic and thermal mechanisms in FUS neuromodulation and highlight the utility of high-frequency, tightly FUS for precise modulation of neuronal activity in brain tissue.

## Data Availability

All data that support the findings of this study are included within the article (and any supplementary files).
